# Retraction: The effect of biosynthesized silver nanoparticles on *Pseudomonas aeruginosa*-infected dogs wounds

**DOI:** 10.1371/journal.pone.0352213

**Published:** 2026-06-23

**Authors:** The PLOS One Editors

Following the publication of this article [1], concerns were raised for Figs 2, 3 and 8. Specifically,

- The following panels appear similar:
  - Fig 2A of this article, Fig 3 in [2], and Fig 2 in [3].
  - Fig 2B of this article, Fig 4 in [2], and Fig 4 in [3].
  - Fig 2C of this article, and Fig 6 in [2].
  - Fig 2D of this article, and Fig 8 in [2].
  - Figs 3A and 3B of this article, and Figs 5 and 7 in [2], respectively.
- There appear to be repetitive areas within one or more panels of figures:
  - Fig 3A.
  - Fig 8.

The corresponding author commented that [2] is a conference paper describing research related to the study described in [1] and that the similarity between these panels is due to the figures presenting the same results. Their response did not resolve the concerns pertaining to the repetitive patterns observed in Figs 3A and Fig 8. The raw image files and individual-level data were not provided for editorial review.

In light of the above unresolved concerns that question the reliability and integrity of the published results in [1], the *PLOS One* Editors retract this article.

MA did not agree with the retraction. AHA, KFH, and AAH either did not respond directly or could not be reached.
